# Modeling the potential range expansion of larger grain borer, *Prostephanus truncatus* (Coleoptera: Bostrichidae)

**DOI:** 10.1038/s41598-019-42974-5

**Published:** 2019-05-03

**Authors:** Frank H. Arthur, William R. Morrison, Amy C. Morey

**Affiliations:** 1 USDA, Agricultural Research Service, Center for Grain and Animal Health Research, 1515 College Ave., Manhattan, KS 66502 USA; 2 Department of Entomology, University of Minnesota, 1980 Folwell Ave., 219 Hodson Hall, St. Paul, MN 55108 USA

**Keywords:** Animal behaviour, Entomology

## Abstract

*Prostephanus truncatus* (Horn) (Coleoptera: Bostrichidae), is a beetle that is a member of a family that is primarily comprised of wood-boring insects, including forest insect pests. It is native to Mexico and Central America, where it has adapted to become a pest of stored maize. It was accidentally introduced into Africa in late 1970s, where it quickly spread throughout the sub-Saharan region, perhaps aided by adaptation to alternate hosts and the ability to persist in non-agricultural habitats. We used the correlative modelling algorithm, MaxEnt, to identify global areas of potential high suitability based on the climate locations with documented populations. Predictions using a model trained in Mexico + Central America showed potential high climatic suitability extending north into the southern United States and southward into South America, including parts of Argentina, but predictions using a model built from African occurrences did not include those areas as highly suitable. However, there was general agreement in both models that large areas of the tropics in the Western Hemisphere and in Asia have climatic conditions that could support *P. truncatus* if it were to become established. The models also showed consistency in capturing potential suitability at sites not used to build a given model. Results can be used as an initial guide to establish surveillance programs to monitor for this insect in high risk areas where it is not currently found, and to proactively mitigate the biosecurity risk from *P. truncatus*.

## Introduction

The larger grain borer, *Prostephanus truncatus* (Horn) (Coleoptera: Bostrichidae), is native to Mexico and Central America, where it is a pest of stored maize though it is also found in non-agricultural habitats^1,2^. However, it was accidentally introduced into east Africa and first documented in Tanzania in the late 1970s^1,3^. It spread rapidly through Central Africa to the west coast, and major outbreaks were soon reported in Ghana and Benin^4,5^. In addition, *P. truncatus* adapted quickly to cassava as an alternate host, and was also able to persist for short periods in non-agricultural habitats such as woodland areas, further facilitating dispersion^6^.

The pest status, spread, and dispersion of *P. truncatus* have been documented in multiple review articles published since the initial introduction^1,6–9^. It has spread throughout the major agricultural regions of sub-Saharan Africa, but most studies involving the sampling of commodities, evaluating the extent of infestations, and determining population ecology were concentrated in Eastern and Western Africa^1,10–12^. A possible reason for this is the presence of international research institutes in those areas, such as branches of the International Institute for Tropical Agriculture in Idaban, Nigeria and Cotonou, Benin, or scientists from the United Kingdom and Germany with extensive research experience in Africa. There is a comparative lack of information for Central Africa, which could be related to political circumstances in that region, as well as the lack of funding and scientists conducting research on stored products and stored product insects.

The invasive *P. truncatus* appears to be more destructive in Africa compared to damage reported in its native range of Mexico and Central America. There are several possible reasons for this increased capacity for damage, including the subsistence-level crib storage systems of maize cobs in Africa, adaptation to dried stored cassava chips as an alternate host, persistence in non-agricultural habitats, and the lack of native natural enemies in the invaded area, including *Teretrius nigrescens* (Lewis) (Coleoptera: Histeridae)^13–17^. However, since the detection of *P. truncatus* in Africa, there have been multiple studies and repeated introductions of this predator on the continent, with varying levels of permanent establishment and success in controlling and limiting *P. truncatus* outbreaks^18–20^.

Given the destructive nature of *P. truncatus* in Africa, knowledge relating to the potential spread of this insect northward or southward from Mexico and Central America and Africa, and eastward from Africa into tropical Asia, as well as knowledge on climatically suitable habitats for invasion would greatly benefit efforts in limiting or eliminating future infestations by targeting detection efforts to areas where the pest is likely to establish. There have been sporadic interceptions of this insect in the United States and Europe, but no true establishment to date. The use of correlative models to estimate species distributions are increasingly viable, in part due to the rise in availability of high resolution climatic and environmental datasets and advances in modelling methods^21,22^. These models identify statistical relationships between locations of species occurrence and a set of environmental predictors, which are then used to identify other areas with similar environmental conditions^23^. The simplicity of the data inputs needed makes correlative models particularly appealing to estimate suitable habitats for invasive species, where often little more information other than records of occurrence are known. However, the extrapolation of correlative model predictions to new areas of space and time – an interest of most applications to invasive species – requires caution^23,24^.

Of the currently available methods, MaxEnt^25^ has shown relatively reliable performance^26^. MaxEnt is a presence-background (or pseudo-absence) model that compares the environmental space at a list of occurrence locations to those at background locations that fall within a user-defined area^27^. The algorithm minimizes the relative entropy between a probability density of the environmental variables at the presence data and another from the background locations, which provides estimates of relative suitability based on the given variables and suitability in geographical space^27,28^. The purpose of the current study is to predict areas for potential range expansion of *P. truncatus* from its current documented distribution. To achieve this, we (1) developed a MaxEnt model based on the documented presence of *P. truncatus* in its native range (Mexico + Central America), (2) developed a second model based on the presence of *P. truncatus* in its introduced range (Africa), and (3) compared the similarity of the two model projections and identified areas of agreement between both models, which may suggest higher risk areas for invasion by *P. truncatus* in natural areas (e.g., areas outside of grain storage facilities).

## Materials and Methods

### Occurrence records and background

A necessary input for correlative species distribution models, including MaxEnt, are geographic coordinates of sites where a species has been documented to occur. An exhaustive literature search was done through various databases, including Agricola, Web of Science, and Google Scholar, to find published, peer-reviewed papers that gave specific names of states, provinces, or regions within a country, including towns or villages, where *P. truncatus* infestations were documented. In some cases, only a general location such as a village or town was listed, others gave a specific sampling area. Geographic coordinates were then obtained through a Google search by listing the location and obtaining information that gave the longitude and latitude. Locations with sporadic sightings or incidental infestations or interceptions were excluded from the list. For many of the references, the sampling sites were one-time locations, with no information on yearly persistence or regularity of infestation but were included in the list because they were in a geographic region where other studies had detected *P. truncatus*, or where *P. truncatus* could potentially survive, based on records of documented establishment in the surrounding geographic region. The literature review yielded multiple, unique geographic coordinates with documented infestations in grain storages or in the surrounding landscape, field crop sites, and forest and woodland sites (Table S1).

Limiting the extent from which MaxEnt draws background locations is recommended to reflect areas accessible to a species or the sampling bias present in occurrence records^28,29^. Given that we had no information on the specific nature of bias in our occurrence sites, we limited background selection to a minimum convex polygon (MCP) with a one-pixel (2.5 arc-minute) buffer drawn around the occurrence points for a given model^29,30^. The package ‘rgeos’^31^ was used to generate the buffered MCPs in R version 3.5.0^32^ and RStudio version 1.1.453^33^. A random sample of 10,000 locations (i.e., the default for MaxEnt) was taken from these reduced areas to serve as background locations for a given model.

### Climate data

Characterizing current and predicted distributions of a species based on climate suitability is widely used^34^ especially with the increasing availability of global climate data. We used 19 gridded temperature and precipitation variables from the WorldClim dataset (v. 2; www.worlclim.org/bioclim)^35^ at 2.5 arc-minute resolution. This dataset contains average monthly global climate data for 1970–2000. The climate rasters were clipped to the extent of a given buffered MCP using the R package ‘raster’ (v. 2.6–7)^36^. To reduce the confounding effects of potential collinearity among climate variables^37^, we then calculated a Pearson correlation coefficient matrix within each MCP space. Only variables with correlations of |*r|* < 0.70 were retained, leaving the final models with 5–6 climate variables (Table 1).

**Table 1:** Environmental variables and parameters used in MaxEnt models of *Prostephanus truncatus*. Environmental variables were from the WorldClim dataset (www.worlclim.org/bioclim) and limited to those with a |*r*| < 0.7 within the area of model development. Regularization and feature combinations were selected for each model based on the lowest AICc value. Resampling method was selected based on sample size and study objective (spatial transferability). Model names refer to the dataset (with *n* occurrences) used to develop the model.

Model	*n*	Environmental variables^a^	Regularization multiplier (β)	Features	Data partitioning method^b^
Mexico + Central America (native range)	32	BIO_3_, BIO_5_, BIO_6_, BIO_12_, BIO_15_	1.5	hinge	*n* − 1 jackknife
Africa (invaded range)	69	BIO_3_, BIO_5_, BIO_6_, BIO_12_, BIO_18_, BIO_19_	1.0	linear, product, quadratic	block

^a^BIO_3_ = Isothermality; BIO_5_ = Max Temperature of Warmest Month; BIO_6_ = Min Temperature of Coldest Month; BIO_12_ = Annual Precipitation; BIO_15_ = Precipitation Seasonality; BIO_18_ = Precipitation of Warmest Quarter; BIO_19_ = Precipitation of Coldest Quarter.

^b^partitioning was done using the R package ‘ENMeval’^43^.

### Direct climate comparisons

For each of the documented occurrences of *P. truncatus*, the climate variable values were extracted and directly compared between populations in Mexico + Central America and Africa to understand how conditions differed between the native and introduced range. The mean value for each population was compared with a *t*-test. Climatic variables compared were those included in one or both final models. These variables included BIO3 (isothermality, percent), BIO5 (maximum temperature of the warmest month, °C), BIO6 (minimum temperature of the coldest month, °C), BIO12 (annual precipitation, mm), BIO15 (precipitation seasonality, percent), BIO18 (precipitation of warmest quarter, mm), and BIO19 (precipitation of coldest quarter, mm). BIO3, isothermality, is derived by calculating the ratio of the mean diurnal range (BIO2) to the annual temperature range (BIO7) and then multiplying by 100. Isothermality quantifies how large the day-tonight temperatures oscillate relative to the summer to-winter (annual) oscillations. Isothermality is generally useful for tropical, insular, and maritime environments^38^. Precipitation seasonality, BIO15, is a measure of the variation in monthly precipitation totals over the course of the year. This index is the ratio of the standard deviation of the monthly total precipitation to the mean monthly total precipitation (also known as the coefficient of variation) and is expressed as a percentage^38^. For all tests, significance was determined at *P* < 0.05.

### Model development and evaluation

Using MaxEnt version 3.3.3k^25^, we built two separate models for *P. truncatus*: one based on occurrences and background from the native range (Mexico + Central America) and one based on occurrences and background from the invaded range in Africa. MaxEnt models made with default settings can result in overfit predictions, so species-specific tuning of complexity is recommended^39–41^. To address this, the regularization multiplier and features classes were selected for each model based on the lowest Akaike Information Criterion corrected for small sample sizes (AICc^42^) using the ‘ENMeval’ R package^43^. ‘ENMeval’ also allows additional methods to partition training and testing data, which we selected based on the recommendations summarized in Muscarella *et al*.^43^. For the model built using native occurrence records, ‘*n* − 1 jackknife’ partitioning was used due to the relatively small number of occurrences (*n* = 32). This method chooses one occurrence point to test a model trained with the remaining (*n* − 1) occurrences, and it was repeated *n* times until each record has been used for testing. For the model built using invaded occurrence records from Africa, the ‘block’ method was used. This method splits approximately equal numbers of occurrence and background points into quadrants based on latitude and longitude.

We quantitatively assessed model performance using the conventional metrics of area under the receiver-operator curve (AUC), which measures the discriminatory ability of each model, and omission rates, which can indicate overfitting. AUC is a threshold-independent performance measure that reflects the probability that a randomly chosen presence site will rank above a randomly chosen background site^25^. Values near 1.0 indicate high discriminatory ability, whereas values of 0.5 (or less) indicate discrimination no better than random^42^. Omission rates (OR) calculate the proportion of test locations with suitability values lower than a specified threshold. The ‘minimum training presence’ threshold omission rate (OR_MTP_) uses the smallest value predicted for any training location, indicating the least-suitable environment where a training point was found. Similarly, the 10% training threshold omission rate (OR_10_) uses the smallest value after excluding the lowest 10% of training suitability values^39,44^. In ideal models, the expected OR_MTP_ and OR_10_ are zero and 10%, respectively, and values higher than expected suggest overfitting. The OR_10_ is less likely to be influenced by extreme/outlier occurrence locations^44^.

### Model projections

Logistic MaxEnt outputs were visualized for each model in ArcMap (ESRI® ArcGIS Desktop 10.6) to show global and region-specific areas of relative suitability between 0–1.0. Models projected into areas with conditions outside of those used to build the model can be unreliable^45^. To identify areas of model extrapolation, multivariate environmental similarity surface (MESS) maps were produced for each MaxEnt model using the package ‘dismo’^46^. The MESS calculates how similar a given point is to a reference set of points for a given climate variable^28^. Values less than zero indicate grids where at least one variable was extrapolated; therefore, we limited each projection space to areas with MESS values of zero or greater to reduce uncertainty (Fig. S1).

To quantify how similar the final spatial projections were, we used ENMTools (v. 1.4.4) to calculate two measures of niche similarity: Schoener’s *D* and *I*, a modified Hellinger distance^47,48^. Both measures compare the suitability values predicted at each grid cell between two models, though *D* assumes the suitability scores are proportional to abundance and *I* does not^48^. Both measures are bound from 0 (no overlap) to 1 (identical), with 0.4–0.6 considered moderate overlap^49^. We also visually identified areas that both models forecasted as suitable, with suitability being defined for each model by the 10^th^ percentile training presence logistic threshold (MTP_10_)^49^. Each model was converted to a binary prediction based on their respective threshold value and then regions that both models forecasted as suitable were isolated using the Intersect (Analysis) tool in ArcMap. The resulting consensus map highlighted areas of higher (suitable in both models) and lower (suitable in only one model) certainty in global suitability predictions. We also performed a Chi-Sq test to compare the suitability classes within each model compared to the null hypothesis that each class would be equally represented.

## Results

### Direct climate comparisons

The climate conditions for recorded occurrences of *P. truncatus* in Mexico + Central America, and Africa were similar for variables BIO3, BIO5, BIO6, and BIO12 (Table 2). BIO15 did not ultimately contribute to the final Mexico + Central America model (Table S2). In the final Africa model, BIO19 and BIO5 showed no permutation importance, but did show some percent contribution (Table S2).

**Table 2:** Summary of climatic variables in the native (Mexico and Central America) and introduced (Africa) range of *P. truncatus*. Means (±SE) are presented for variables provided to one or both of the models. An asterisk indicates a significant (*P* < 0.05) difference between the regions for a given variable if it was used in both final models.

Climate Variable^a^	Mexico and Central America	Africa	*t*	*P*
	Mean ± SE	Mean ± SE		
BIO3	203.9 ± 4.8	220.4 ± 4.6	1.97	0.05
BIO5	229.2 ± 11.4	241.9 ± 11.1	0.64	0.52
BIO6	12.0 ± 1.8	9.8 ± 1.3	0.83	0.41
***BIO12***	***129.5*** ± ***3.2***	***118.1*** ± ***2.3***	***2.44***	0.02*
BIO15	332.6 ± 4.5	—	—	—
BIO18	—	245.2 ± 3.2	—	—
BIO19	—	232.4 ± 5.4	—	—

^a^For a definition of each variable and associated units, please see the materials and methods.

### Assessment of Mexico + Central America model and global predictions

The model trained in Mexico + Central America showed a high degree of discriminatory ability based on the AUC, and closely met the expected value for the omission rate (AUC and OR_MTP_, see Table 3). The AUC_diff_ and OR_10_ values for the model suggests that there is some degree of overfitting for the model (Table 3). The Mexico + Central America-trained model ultimately included four uncorrelated variables from the WorldClim dataset, namely BIO3, 5, 6, and 12.

**Table 3:** Evaluation and similarity metrics for MaxEnt models of *Prostephanus truncatus*. Metrics were generated in R (‘ENMeval’^43^ and ENMTools^48^. Similarity indices (Schoener’s D and *I*) compared the projection areas remaining after MESS exclusion (see methods) between the two models.

Model	AUC_TEST_^a^	AUC_DIFF_^b^	OR_MTP_^c^	OR_10_^d^	MTP_10_^e^	Schoener’s *D*	*I*
Mexico + Central America (native range)	0.751	0.116	0.031	0.219	0.342	0.606	0.883
Africa (invaded range)	0.593	0.179	0.059	0.294	0.216		

^a^AUC_TEST_ measures the ability of the model to distinguish the occurrence points used in model testing from background points with 1.0 being perfect discrimination.

^b^AUC_DIFF_ is the difference between the AUC using test and training occurrences; high differences indicate model overfitting.

^c^OR_MTP_ is the omission rate for the proportion of test locations with suitability values lower than the smallest value predicted for any training location (minimum training presence; MTP); overfitting is indicated by deviations from the expectation of zero.

^d^OR_10_ is the omission rate for the proportion of test locations with suitability values lower than the smallest value after excluding the lowest 10% of training suitability values (10% training omission rate); overfitting is indicated by deviation from the expectation 0.10.

^e^MTP_10_ is the minimum predicted logistic value for the training sites after excluding the lowest 10% of training site values.

**Figure 1:**
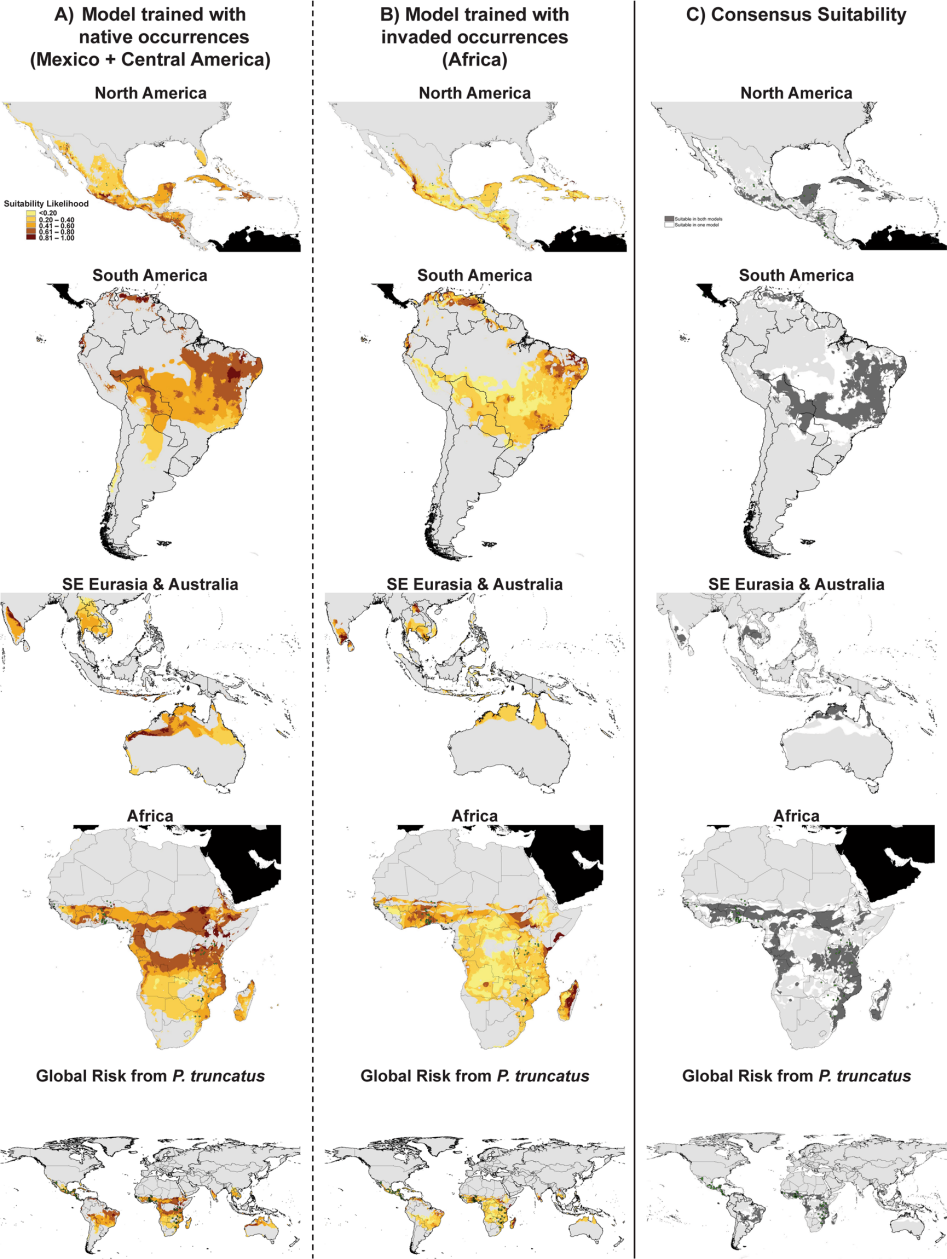
Forecasted geographic suitability of *Prostephanus truncatus* based on temperature and moisture. The first two columns show the predicted suitability projected in various regions from two different MaxEnt models: (**A**) a model trained using occurrence and background locations from the native range of *P. truncatus* in Mexico + Central America and, (**B**) a model trained using occurrence and background locations from the invaded range of *P. truncatus* in Africa. The third column (far right) shows regions where the two models overlap (dark grey) in projected suitability based on their respective 10% minimum training thresholds (Table 3). Light grey areas in all maps indicate areas removed from model projection based on MESS (see Materials and Methods and Fig. S1). Green dots are documented records of occurrence (see Table S1).

As expected, the Mexico + Central America-trained model showed that all the recorded occurrences of *P. truncatus* in its native range were highly suitable for potential establishment and population development (Fig. 1A). In addition, there are areas in Mid- and Southern California, most of Florida, and large sections of the Caribbean that appear climatically highly suitable for colonization by *P. truncatus* according to this model.

In South America, large areas of Brazil and the northern regions of Argentina, Venezuela, much of Bolivia, and western Paraguay appear highly suitable (>0.80) for this species (Fig. 1A). In Southeast Asia, the most probable areas of suitability are western and southern India, large sections of Myanmar, Thailand, Cambodia, Vietnam, Indonesia, and in sporadic locations in the Philippines. Finally, large sections of northern Australia in a belt stretching from the eastern to western coasts of the continent also appear highly suitable for establishment of *P. truncatus*. Much of equatorial Africa from 12°N to −20°S, except for a large area of central Democratic Republic of Congo is considered a climatically highly-suitable habitat for *P. truncatus* according to the Mexico + Central America-trained model. The majority of the documented occurrences in Africa (55%) were predicted by the Mexico + Central America-trained model.

### Assessment of the African model and global predictions

The second model was trained on the recorded occurrences of *P. truncatus* from the invaded range in Africa. This model showed a moderate degree of discriminatory ability based on the AUC (Table 3). Notably, there were several recorded occurrences in the native range that the African-trained model did not predict as potential suitable areas, but 68% of the occurrences were still predicted by the Africa-trained model (Table 5). Further, this model had a higher AUC_diff_ value and omission rate than the Mexico + Central America-trained model, with a OR_MTP_ roughly near zero, but an OR_10_ rate near 30% (Table 3), indicating some degree of overfitting. The Africa-trained model included six variables instead of four, namely BIO3, 5, 6, 12, 18, and 19 (Table 1).

Overall, fewer areas within our projection space were predicted as highly suitable in the African-trained model (>0.80 suitability; Table 4, Fig. 1B). In addition, overall there was also more land area considered to have low suitability (<0.21 suitability) for *P. truncatus* in the African model compared to the Mexican + Central American model.

**Table 4:** Areas of suitability for *P. truncatus* as predicted by the Mexico + Central America and African models.

Model training region	Suitability Class	Area (km^2^)	Proportion of total projection area^a^
Mexico + Central America	<21	376,379	0.013*
	21–40	8,426,985	0.281
	41–60	12,285,599	0.410*
	61–80	7,803,100	0.260
	81–100	1,066,489	0.035
	Total	29,958,552	
Africa	<21	4,505,269	0.170
	21–40	15,324,383	0.577*
	41–60	4,389,318	0.165
	61–80	1,844,947	0.069*
	81–100	513,238	0.019*
	Total	26,577,154	

Suitability class, area, and proportion of total global projection area are listed for each model. Expected proportion of total projection area was assumed to be 0.2 for each class within each model under the null hypothesis. Significant deviations from the null hypothesis are denoted by an asterisk (χ^2^-test, Bonferroni correction). For both models, there were no areas predicted as 0 suitability within our areas of projection.

^a^Total area where overlap between the two models forecasted suitability >0.0 was 18,509,504 km^2^. The proportion of the Africa model in agreement with the Central America model was 0.70. The proportion of the Central America model in agreement with the Africa model was 0.62.

With regards to North America, most areas in the United States, were outside the range of our data, but Mexico the Caribbean Cuba, most of Haiti and the Dominican Republic, and Jamaica and Puerto Rico were considered potential areas of establishment (Fig. 1B). Similar to the Mexico + Central American-trained model, large areas of Brazil, Bolivia, Paraguay, and northern Venezuela were considered climatically highly-suitable for establishment of *P. truncatus* by the African-trained model, though not northern Argentina. In southeastern Eurasia and Oceana, Thailand, Cambodia, Vietnam, southern India, and northern Laos were considered highly suitable by the model, with sporadic high climatic suitability in the Philippines and Indonesia. Similar to the Mexican + Central American-trained model, the African-trained model considered regions of northern Australia climatically suitable for *P. truncatus*. Much of equatorial Africa was also considered to be climatically highly-suitable by the African-trained model, and included all the recorded occurrences of the species, but also similarly predicted less climatic suitability in the central Democratic Republic of Congo.

### Assessment of dual model consensus and global predictions

There was moderate to strong overlap in areas considered climatically highly-suitable by the models depending on whether Schoener’s *D* or *I* is considered. Both models assessed much of Western and Southern Mexico, as well as much of Central America, and the larger Caribbean islands as potential habitats for establishment and dispersal of *P. truncatus*, though some of the recorded occurrences fell outside the consensus zones in the native range (Table 5). In South America, consensus zones of climatic suitability included large areas of Brazil, Bolivia, western Paraguay, and coastal Venezuela. Both models overall predicted that much of tropical Africa was highly suitable for *P. truncatus*. In Eurasia and Oceana, both models agreed that most of Cambodia, northern Laos, large areas of Thailand, southern India, sporadic locations in Indonesia, and northern Australia were moderately suitable (0.40 to 0.80) areas for *P. truncatus*. Both models overall predicted that much of tropical Africa was highly suitable for *P. truncatus*.

**Table 5:** Counts of occurrence records for *Prostephanus truncatus* (see Table S1) in relation to various categories of predicted suitability in each model. See Material and Methods for full description of each model.

Model	Occurrence record dataset													
	Mexico/Central America (n=32)							Africa (n=69)						
	Within modeled suitability space*	*Outside modeled suitability space*	<*0.21*	*0.21–0.40*	*0.41-0.60*	*0.61–0.80*	*0.80–1.00*	*Within modeled suitability space*	*Outside modeled suitability space*	<*0.21*	*0.21–0.40*	*0.41–0.60*	*0.61–0.80*	*0.80–1.00*
Africa	22	10	6	14	1	1	0	69	0	7	28	13	14	7
Mexico + C. America	32	0	0	7	9	15	1	38	31	1	13	9	14	1
Consensus	16	16	n/a	n/a	n/a	n/a	n/a	24	45	n/a	n/a	n/a	n/a	n/a

*The suitability space for the binary Consensus model was considered here as those areas that both models predicted suitable, based on their respective 10 percent minimum training presence logistic threshold (i.e., dark grey areas in Fig. 1c). The suitability space for the remaining two models were those areas with a continuous suitability value >0 (i.e., colored areas in Fig. 1a-b).

## Discussion

Our MaxEnt models utilized four to six different climatic variables to predict areas where *P. truncatus* could potentially establish outside of storage facilities, based on either the current distribution in Mexico + Central America or in Africa. The two models ultimately were based on similar climate variables, with the Africa model using two additional variables (i.e., BIO18 and BIO19). Of the shared variables, only the amount of annual precipitation (i.e., BIO12) differed significantly between the two modelled regions. However, there were some other differences between the two models, in that the Mexican + Central American model identified sites in the southern US that were potential areas of high suitability (i.e., >0.8), while the African model did not. In the southern US, maize is typically harvested in late July and early August, and population prediction models coupled with historical weather data show that the warm temperatures will support rapid population growth of other stored product species, such as the maize weevil, *Sitophilus zeamais* (Motschulsky), even with the use of aeration to cool the grain mass and modify storage bin temperatures^50^.

The African model was perhaps more conservative, but both models identified areas in the Caribbean and areas in tropical and sub-tropical South America as areas of high climatic suitability. In general, though there were some zones of disagreement, both models showed significant overlap with each other in areas designated as climatically suitable (Fig. 1C). Importantly, for both models, we conservatively chose to limit the areas of prediction to those regions that did not require extrapolation beyond the range of our data (i.e., light grey areas in Fig. 1). This decision was intended to reduce uncertainty associated with extrapolation^23^ and does not indicate that these areas are not at risk. There are three possible explanations for what may be happening in the grey areas of our projections: (1) they could truly be outside the climatic tolerances for this species, (2) they could be outside the current realized niche of the species, and/or (3) our occurrence datasets may be incomplete in representing the current niche of the species and additional records may change the extent of these areas. It is possible that *P. truncatus* may be able to survive inside a buffered anthropogenic structure such as a grain bin or food facility where temperature effects are mitigated.

Network mapping showing routes of trade and their relative frequency with ports in the native and invaded ranges of *P. truncatus* may help illuminate relative risk in these grey areas. For example, Paini and Yemshanov^51^ used the international marine shipping network centered around Australia to predict the relative risk of entry by the quarantine threat posed by the, khapra beetle, *Trogoderma granarium* Everts (Coleoptera: Dermestidae), and found ships arriving from Taiwan and the Republic of Korea were the most likely sources for entry of the species into the country. Moreover, it is also possible that global climate change may shift the range of suitable areas northward and southward towards the poles for *P. truncatus*, as has been reported for other insect species^52,53^. This may have repercussions for the stored product species community ecology in newly invaded areas, with *P. truncatus* likely dominating on maize, and causing correspondingly more damage^54^.

Both models consistently identified tropical and sub-tropical areas as climatically highly-suitable for establishment of *P. truncatus*. This is important because cassava is a primary crop in the tropics and subtropics, and many of the publications documenting the rapid spread and dispersal of *P. truncatus* in Africa cite the species’ quick adaptation to cassava as an alternate agricultural host as a primary contributing factor^15,55^. In Africa, infestations often occur when cassava is stored as dried chips. Cassava is native to South America and is now grown throughout Brazil, in tropical Africa and Asia, in India and China, and parts of tropical Australia^56^. It is used as a staple food because of its high starch content in the roots, as well as in industrial processes^57^. Production for human food is increasing and some researchers have stated that cassava now ranks behind wheat, corn, and rice in global production and utilization^58^. Although potential spread of *P. truncatus* might be limited because cassava is cultivated in large plantations in South America and tropical Asia, and may not be stored as dried chips, it is still reasonable to assume that if *P. truncatus* was introduced into areas where cassava is grown and where *P. truncatus* has not been established, some adaptation and dispersal could still occur.

Many studies in Africa also document survival and reproduction of *P. truncatus* on woody host material, including live and dead branches, branches girdled by attacking wood-boring insects, or on seeds^6,59,60^. Though listings for specific species can be inconsistent and contradictory, there are obviously some woody species that will support development and reproduction in addition to incidental or low-level attack and survival. Nansen *et al*.^59^ reported reproduction on teak (*Tectona grandis* L.) seeds. The natural range of teak is the Indian peninsula and Southeast Asia, but it is also planted in managed plantations throughout the tropics^61^. While there is no evidence that *P. truncatus* would attack mature trees, it seems that it could indeed survive and reproduce on dead branches and seeds and serve as an alternate host as well. Since *P. truncatus* is a member of the family Bostrichidae, and most members of this family are wood-boring insects, it is not surprising that it can potentially survive and reproduce on woody material. Both the Mexican + Central American and African models predict that *P. truncatus* could survive in tropical areas where teak and cassava are both present. It is also possible that other woody species could serve as alternate hosts as well.

The MaxEnt modeling approach performs particularly well with small samples size^59^ and has been utilized for a variety of invasive species, including the brown marmorated stink bug (*Halyomorpha halys* [Stål])^62^, the emerald ash borer (*Agrilus planipennis* Fairmaire)^63^, and spotted wing drosophila (*Drosophila suzukii*) Matsumura^64^. There were some discrepancies between our two models but predicted climatic suitability can differ between models based on native versus invaded populations for multiple reasons, such as shifts in climatic niche with invasion^65,66^, other biotic sieves such as interspecific competition that limit the realized niche^67^ or an incomplete description of climatic suitability in the invaded range due to lack of equilibrium with the environment^23^. We compared the two models using two indices of similarity, Schoener’s *D* and *I*, a modified Hellinger distance^45,47^. Others have found that *I* tends to estimate greater similarity than Schoener’s *D*^48,68^, and that *D* may be better suited for computing niche overlap^48^.

In conclusion, our modeling results provide guidelines for predicting potential geographic regions where *P. truncatus* could survive outside of storage facilities or in unbuffered environments, and consequently, the increased risk of invasion into storage facilities due to populations sustained outside these facilities. Our models suggest grains stored in tropical Asia may be especially at risk if *P. truncatus* were to become established in the surrounding environment. This modeling approach could be used to guide directed studies on temperature tolerance for management in storage facilities. These results emphasize the need for monitoring of this pest in tropical areas where it is not currently present or established, and for governments and research institutions to be proactive in their surveillance and detection efforts^69^.

## Supplementary information

Supplementary Table and Figure

## Data Availability

All data on which the model in this paper was based are available as Supplemental Files.
