# Supplementary material for: Modeling the potential range expansion of larger grain borer, Prostephanus truncatus (Coleoptera: Bostrichidae)
Source: Sci Rep. 2019 May 3;9:6862. doi: 10.1038/s41598-019-42974-5 (PMC6499817; doi:10.1038/s41598-019-42974-5)

Title Page for Supplementary Tables and Supplemental Figure

Modeling the potential range expansion of larger grain borer, *Prostephanus truncatus* (Coleoptera: Bostrichidae)

Frank H. Arthur[1*], William R. Morrison III[1], and Amy C. Morey[2]

[1] USDA, Agricultural Research Service, Center for Grain and Animal Health Research, 1515 College Ave., Manhattan, KS 66502

[2] Department of Entomology, University of Minnesota, 1980 Folwell Ave., 219 Hodson Hall, St. Paul, MN 55108

[*] Corresponding author's email: frank.arthur@ars.usda.gov

Supplementary File for Arthur et al. 2018.

Supplementary Table S1. Locations and geographic coordinates of sites in Mexico, Central America, and Africa where infestations of *Prostephanus truncatus* were documented either in unbuffered grain storages or in non-agricultural habitats (excludes incidental occurrences/interceptions outside of normal range).

Country	Region	Latitude	Longitude	Reference
Mexico				
	Campache	18.9312 N	90.2618 W	[1]
	Chiapas	16.7569 N	93.1292 W	
	Guerrero	17.4395 N	99.5451 W	
	Morelos	18.6613 N	99.9013 W	
	Oaxaca	17.0542 N	96.7312 W	
	Quintana Roo	18.1818 N	88.4791 W	
	Tabasco	17.8409 W	92.6189 W	
	Tamaulipas	24.6669 N	98.8363 W	
	Yucatan	20.7079 N	89.0943 W	
	Chilcauautla	20.3277 N	99.2314 W	[2]
	El Baijo	16.8891 N	95.2177 W	[3]
	Guanajuato	21.0190 N	101.2574 W	[4]
	Jalisco	20.6595 N	103.3494 W	
	La Laguna	21.3905 N	101.3612 W	
	Veracruz	19.1738 N	96.1432 W	[5]
	Hermosillo	29.8056 N	109.6791 W	
	Moctezuma	28.7630 N	109.6346 W	
	Obregon	29.0730 N	110.9559 W	
Central America	Soyopa	27.4828 N	109.9304 W	

Honduras				
	Yoro	15.2950 N	87.1423 W	[6]
	Jamastran Valley	14.0000 N	86.4137 W	
	Zamarato valley	14.0423 N	87.1921 W	[1]
Nicaragua				
	Managua	12.1150 N	86.8362 W	[7]
	Matagalpa	12.9290 N	85.9151 W	
Costa Rica				
	Canafistula	10.2830 N	85.7830 W	[8]
	La Delicas	10.7000 N	85.3830 W	
	Pargos	10.2026 N	85.8256 W	
	Pariso	9 .8392 N	83.8664 W	
	Pinilla	10.2450 N	85.3811 W	
	San Blas	9.8640 N	85.1830 W	
	Sardinal	10.5174 N	85.6475 W	
Africa				
Benin				
	Abney-Calavi	6.4485 N	2.3468 E	[9]
	Dogbo	6.8050 N	1.7880 E	
	Bante	9.4112 N	1.8879 E	[10]
	Malanville	11.8633 N	3.3842 E	[11]
	Lokossa	6.6448 N	1.7198 E	[12]
Burkina Faso				
	Sanga	11.0591 N	0.6241 E	[13]
Ghana				
	Ho	6.6101 N	0.4785 E	[14]

	Hohoe	7.1519 N	0.4738 E	
	Kpando	7.4055 N	0.4597 E	
	Jasikan	6.9906 N	0.2929 E	
	Ejura	9.4034 N	0.8424 W	[15,16]
	Kumasi	6.6666 N	1.6163 W	
	Tamale	7.3847 N	1.3590 W	
Kenya				
	Jenda	10.7500 S	33.9333 E	[17]
	Kiambu	1.0388 S	37.0834 E	
	Murang'a	0.6695 S	37.0621 E	
	Nakuru	0.3030 S	36.0800 E	
	Nyeri	0.3935 S	37.0025 E	
Malawi				
	Mchenachena	12.3616 S	36.5560 E	[18]
Mozambique				
	Almada	18.5807 S	33.1918 E	[19]
	Cruz	18.1807 S	33.1159 E	
	Honde	18.1929 S	33.1159 E	
	Mozambiza	18.1954 S	33.1036 E	
	Mucombezi	18.4000 S	33.1853 E	
	Mutarara	17.4431 S	35.0737 E	
	Punge-South	18.3326 S	33.1614 E	
	Vanduzi	18.5727 S	33.1603 E	
Niger				
	Dosso	13.0505 N	3.2081 E	[20]
	Gaya	11.8843 N	3.4549 E	
	Naimey	13.5116 N	2.1254 E	
Nigeria				

	Benue	6.4584 N	7.5464 E	[21]
	Inugu	7.3508 N	8.8363 E	
	Ibadan	7.3775 N	3.9470 E	
Rwanda				
	Makagne Sory	12.5435 N	14.5701 W	[22]
Senegal				
	Velingara Boydo	12.5057 N	15.0236 W	[23]
South Africa				
	Kruger Park	23.9884 S	30.8129 E	[24]
Tanzania				
	Maragoro	6.8278 S	37.6591 E	[25]
	Mwanza	2.5164 S	32.9175 E	
	Kihama	3.8376 S	32.5938 E	
	Kilosa	6.8343 S	36.9971 E	
	Nyalikungu	3.1848 S	33.7797 E	
	Shinyanga	3.6891 S	33.4271 E	
Togo				
	Amlame	7.4562 N	0.9033 E	[26]
	Aneho	6.2273 N	1.5814 E	
	Atakpame	7.5287 N	1.1305 E	
	Badou	7.5866 N	0.6086 E	
	Bafilo	9.3568 N	1.2483 E	
	Bassar	9.2661 N	0.7771 E	
	Dapaong	10.8733 N	0.2010 E	
	Kpalime	6.9463 N	1.1689 E	
	Mango	10.3551 N	0.4738 E	
	Notse	6.9463 N	1.1689 E	
	Sokode	8.9780 N	1.1449 E	

	Tchamba	9.0295 N	1.4169 E	
	Tsevie	6.4239 N	1.2073 E	
	Vogan	6.3337 N	1.5281 E	
<u>*Uganda*</u>				
	Busia	0.4707 N	34.0429 E	[22]
Zambia				
	Chadiza	14.0636 S	32.4382 E	[18]
	Katete	14.0584 S	32.0440 E	
	Mugabe	13.1404 S	27.8493 E	
Zimbabwe				
	Mashonaland West	17.2847 S	29.9741 E	[24]
	Mashonaland East	18.7557 S	31.8795 E	

References for S1 Table

1. Rivera, R. R. The larger grain borer in southern Mexico. Biological Control of the Larger Grain Borer, (Markham, R. H., & Herren, H. R., eds), pp. 25-29, *Proceedings of an IFTA/FAO Coordination Meeting*, Cotonou, Benin (1990).

2. Rios Ibarra, R. M., Markham, R. H., Novillo-Rameix P., & Wright, V. F. Ecology and biological control of the Larger Grain Borer in Mexico and Honduras. Implementation of and Further Research on Biological Control of the Larger Grain Borer (Bocye, J., Wright, M. & Laborius, G. A., eds.), pp. 123-136, *GTZ Publications*, Eschborn, Germany (1992)

3. Tigar, B. J., Key, G. E., Flores, M. E., & Vazquez. A. Field and post-maturation Infestation of Maize by Stored Product Pests in Mexico. *J. Stored Prod. Res.* 30, 1-8 (1994a),

4. Tigar, B. J., Osborne, P. E., Key, G. E., Flores, S. E., & Vazque, A. Insect pests associated with rural maize stores in Mexico with particular reference to *Prostephanus truncatus* (Coleoptera: Bostrichidae). *J. Stored Prod. Res.* 30, 267-281 (1994b).

5. Corral, F. W., & Cortez-Rocha, M. O. Presence of *Prostephanus truncatus* (Horn) (Coleoptera: Bostrichidae) in Sonora, Mexico, first report. *Southwest. Entomol.* 26, 151-158 (2001).

6. Cave, R. D., de Malo, V. W. Larger grain borer research perspectives in Central America. Biological Control of the Larger Grain Borer, (Markham, R. H. & Herren, H. R., eds) pp. 30-34, *Proceedings of an IFTA/FAO Coordination Meeting*, Cotonou, Benin, (1990)

7. Giles, P.H. & Leon, O.J. Infestation problems in farm-stored maize in Nicaragua. pp. 68-76, *Proceedings of the First International Working Conference on Stored Product Protection*, Savannah, GA, USA (1975).

8. Bocye, J. Ecological aspects of *Prostephanus truncatus* (Horn) (Col.: Bostrichidae) in Central America. In Biological Control of the Larger Grain Borer, (Markham, R. H. & Herren, H. R., eds), pp. 73-86, *Proceedings of an IFTA/FAO Coordination Meeting*, Cotonou, Benin, (1990).

9. Meikle, W. G., Holst, N., Cholz, D., & Markham, R. H. Simulation model of *Prostephanus truncatus* (Coleoptera: Bostrichidae) in rural maize stores in the Republic of Benin. *Environ. Entomol.* 27, 59-69 (1998).

10. Borgemeister, T. C., Adda, C., SeÂtamou, M., Hell, K., Djomamou, B., Markham, R. H., Cardwell, K. F. Timing of harvest in maize: effects on post harvest losses due to insects and fungi in central Benin, with particular reference to *Prostephanus truncatus* (Horn) (Coleoptera: Bostrichidae). *Agric. Ecosyst. Environ.* 69, 233-242 (1998).

11. Bakoye O. N., Baoua, L. B., Seyni, H., Amadou, L., Murdock, L. L, & Baributsa, D. Quality of maize for sale in markets in Benin and Niger. *J. Stored Prod. Res*. 71, 99-105 (2017).

12. Borgemeister, C., Tchabi, A., & Scholz, D. The origin of migrating *Prostephanus truncatus* collected in different ecological habitats in southern Benin. *Entomol. Exp. Appl.* 87, 285-294 (1997).

13. Muatinte, B. L., Van Den Berg, J., & Santos, L A. *Prostephanus truncatus* in Africa: a review of biological trends and perspectives on future pest management strategies. *Afr. Crop Sci. J.* 22, 237-256 (2014).

14. Addo, S., Birkinsaw, L. A., & Hodges, R. J. Ten years after the arrival in Ghana of the Larger Grain Borer: Farmer's responses and adoption of IPM strategies. *Int. J. Pest Manage.* 48, 315-325 (2002).

15. Danso, J. K., Osekre, E. A., Opit, G. P., Manu, N., Armstrong P., Arthur F. H., Campbell, J. F., & Mbata, G. Moisture content, insect pests and mycotoxin levels of maize at harvest and post-harvest in the Middle Belt of Ghana. *J. Stored Prod. Res.* 74, 46-55 (2017).

16. Danso, J. K., Osekre, E. A., Opit, G. P., Manu, N., Armstrong, P., Arthur, F. H, Campbell, J. F., Mbata, G., & McNeill, S. G. Post-harvest insect infestation and mycotoxin levels in maize markets in the Middle Belt of Ghana. *J. Stored Prod. Res.* 77, 9-15 (2018).

17. Njoroge, A.W., Affognon, H., Mutungi, C., Rohde, B., Richter, U., Hensel, O., & Mankin, R. W. Acoustic survey in Kenya's grain storage facilities and the promise of acoustic detection of pests. pp 121-123,*1st All Africa Postharvest Conference, special edition,* CRC Press, Taylor and Francis, Abington, UK, (2017).

18. Kamanula,, J., Sileshi,, G. W., Belmain, S. R., Sola, P., Mvumi, B., Nyirenda, G. K. C., Nyirenda, S. P. & Stevenson, P. C. Farmers' insect pest management practices and pesticidal plant use in the protection of stored maize and beans in Southern Africa. *Int. J. Pest Manage.* 7, 41-49 (2010).

19. Muatinte, B. L.& Cugala, D.R. Monitoring the establishment and dispersal of *Teretrius nigrescens* Lewis (Coleoptera: Histeridae), a predator of *Prostephanus truncatus* Horn (Coleoptera: Bostrichidae) in Manica Province, Mozambique. *Afr. Entomol.* 23, 250-254 (2015).

20. Adda, C., Borgemeister, C., Meikle, W. G., Markham, R. H, Olaleye, I., Abdou, K. S.& Zakari,, M. O. First record of the larger grain borer, *Prostephanus truncatus* (Coleoptera: Bostrichidae), in the Republic of Niger. *Bull. Entomol. Res.*, 83-85 (1996).

21. Nwankwo, E. N, Okonkwo, N. J., & Egwuatu, R. I. Length of exposure and quantal responses of four strains of *Prostephanus truncatus* (Horn) (Coleoptera: Bostrichidae) strains to pyrethroid insecticides. *Int. J. Sci. Technol.* 2, 147-154 (2014).

22. Muatinte, B.L., Van Den Berg, J., & Santos, L.A. 2014. *Prostephanus truncatus* in Africa: a review of biological trends and perspectives on future pest management strategies. *Afr. Crop Sci. J.* 22, 237-256 (2014).

23. Gueye, M. T., Goergen, G., Badiane, D., Hell, K., & Lamboni, L. First report on occurrence of the larger grain borer *Prostephanus truncatus* (Horn) (Coleoptera: Bostrichidae) in Senegal. African Entomol. 16, 309-311 (2008).

24. Anonymous. First record of *Prostephanus truncatus* in South Africa. *EPPO Reporting Service* no. 06. (1999)

25. Hodges, R. J., Dunstan, W. R., Magazini, I., & Golob, P. An outbreak of *Prostephanus truncatus* (Horn) (Coleoptera: Bostrichidae) in east Africa (Tabora, Tanzania). *Prot. Ecol.* 5, 183-194 (1983).

26. Mutlu, P. Ability of the predator *Teretriosoma nigrescens* Lewis (Col.: Histeridae) to control larger grain borer *Prostephanus truncatus* (Horn) (Col.: Bostrichidae) under rural storage conditions in the southern region of Togo. Proceedings of the 6th International Working Conference on Stored Product Production, (Highley, E., Wright, E.J., Banks, H.J., & Champ, B. R, eds.), pp. 1116-1121, *CABI* Canberra, Australia (1994).

27. Admire, M. T & Tinase, S. Efficacy of traditional and improved granaries in protecting maize from *Prostephanus truncatus* in small holder farming storage system in Makonde District, Zimbabwe. *World J. Agric Res* 2*,* 63-69 (2014)

Supplementary File for Arthur et al. 2018.

Supplementary Table S2: Summary of the relative contributions of each WorldClim climate variable input into the MaxEnt models of *Prostephanus truncatus*. Models are named for the regions of occurrence upon which they are based -- the native (Mexico and Central America) and introduced (Africa) range of *P. truncatus*. See Phillips (2017) for further description of the calculations and the Materials and Methods section of the main text for climate variable description.

Reference for S2 Table

Phillips, S.J. A Brief Tutorial on Maxent. (2017) Available from url: http://biodiversityinformatics.amnh.org/open_source/maxent/. Accessed on 2019-1-6.

**Model**	**Variable**	**Percent Contribution**	**Permutation Importance**
Mexico/Central America	BIO 3	25.19	17.87
	BIO 5	20.38	13.46
	BIO 6	45.71	58.69
	BIO 12	8.72	9.99
	BIO 15	0.00	0.00
Africa	BIO 3	17.37	2.28
	BIO 5	11.12	0.00
	BIO 6	18.51	42.54
	BIO 12	15.94	25.75
	BIO 18	33.60	29.44
	BIO 19	3.48	0.00

Supplemental Figure S1. **Global MESS maps.** Blue shades indicate MESS values >0 (i.e. climate variables are within the range of those used to build the model), and red shades indicate values <0 (i.e. at least one climate variables has a value outside the range of those used to build the model).

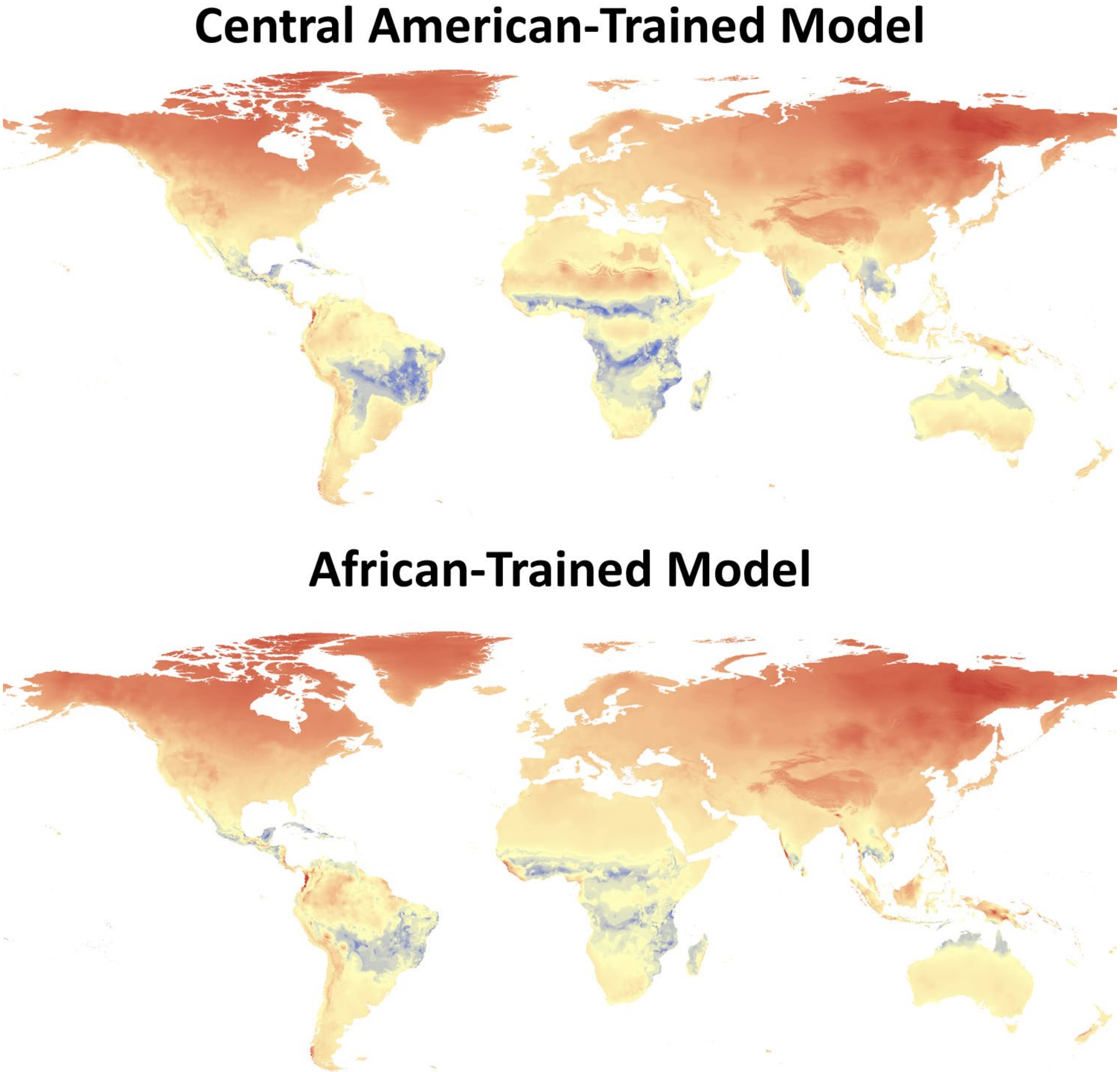

Supplement: Supplementary file 1 — Supplementary Table and Figure [file 41598_2019_42974_MOESM1_ESM.pdf]
